# A Rare Early-Onset Fatal Complication after Transarterial Chemoembolization: A Case Report and Review of the Literature

**DOI:** 10.3390/curroncol31040147

**Published:** 2024-04-03

**Authors:** Monika Péčová, Jakub Benko, Martin Jozef Péč, Jakub Jurica, Simona Horná, Tomáš Bolek, Tatiana Hurtová, Ján Sýkora, Kamil Zeleňák, Matej Samoš, Juraj Sokol

**Affiliations:** 1 Department of Hematology and Transfusiology, Jessenius Faculty of Medicine in Martin, Comenius University in Bratislava, 036 59 Martin, Slovakia; kucerikova8@uniba.sk (M.P.);; 2 Oncology Centre, Teaching Hospital Martin, 036 59 Martin, Slovakia; 3 Department of Internal Medicine I., Jessenius Faculty of Medicine in Martin, Comenius University in Bratislava, 036 59 Martin, Slovakia; benko36@uniba.sk (J.B.); bolek2@uniba.sk (T.B.);; 4 Department of Cardiology, Teaching Hospital Nitra, 949 01 Nitra, Slovakia; 5 Department of Cardiology, Teaching Hospital Trenčín, 911 71 Trenčín, Slovakia; 6 Department of Infectology and Travel Medicine, Jessenius Faculty of Medicine in Martin, Comenius University in Bratislava, 036 59 Martin, Slovakia; 7 Department of Radiology, Jessenius Faculty of Medicine in Martin, Comenius University in Bratislava, 036 59 Martin, Slovakia; 8 Division of Acute and Interventional Cardiology, Department of Cardiology and Angiology II, Mid-Slovakian Institute of Heart and Vessel Diseases (SÚSCCH, a.s.) in Banská Bystrica, 974 01 Banská Bystrica, Slovakia

**Keywords:** hepatocellular carcinoma, transarterial chemoembolization, rupture

## Abstract

Transarterial chemoembolization (TACE) is a minimally invasive treatment for liver cancer, often employed as a bridging therapy or destination treatment for non-operable cases. This case report discusses an 82-year-old woman with a large hepatocellular carcinoma (HCC) who underwent elective TACE due to the high surgical risk associated with her tumor size. Unexpectedly, the patient experienced liver rupture 20 h post-procedure, leading to acute surgical intervention. Despite successful hemostasis during surgery, the patient succumbed to progressive multi-organ failure. We aimed to search the PubMed database for documented cases of ruptured HCC after TACE. This study highlights risk factors for spontaneous HCC rupture and specific factors associated with TACE-induced rupture. Transarterial embolization (TAE) is currently favored as the treatment method for spontaneous ruptures, while the optimal therapy for TACE-induced ruptures remains unclear. In conclusion, this case underscores the importance of recognizing the rare complication of HCC rupture post-TACE and the need for personalized risk assessment. While TAE emerges as a primary treatment choice, the lack of consensus necessitates further studies to establish evidence-based approaches for managing this uncommon yet life-threatening complication.

## 1. Introduction

Transarterial chemoembolization (TACE) is an advantageous, less invasive method for the treatment of liver cancer, mainly as bridging therapy to liver transplantation or destination therapy for non-operable cases [1]. This method is applicable to not only hepatocellular carcinoma (HCC) but also secondary tumors, most commonly colorectal, neuroendocrine, and melanoma metastases. During the procedure, the supplying artery of the tumor is catheterized, and chemoembolization agents are administered to decrease its size or even cure the tumor. The most typical complication requiring intervention is acute cholecystitis. TACE is also associated with a small risk of bleeding, infection, and kidney injury. Complications are uncommon and usually well tolerated. An infrequent consequence is the rupture of the tumor with ensuing bleeding [2]. We describe a case of liver rupture after TACE with subsequent surgical intervention.

## 2. Case Report

**Figure 1:**
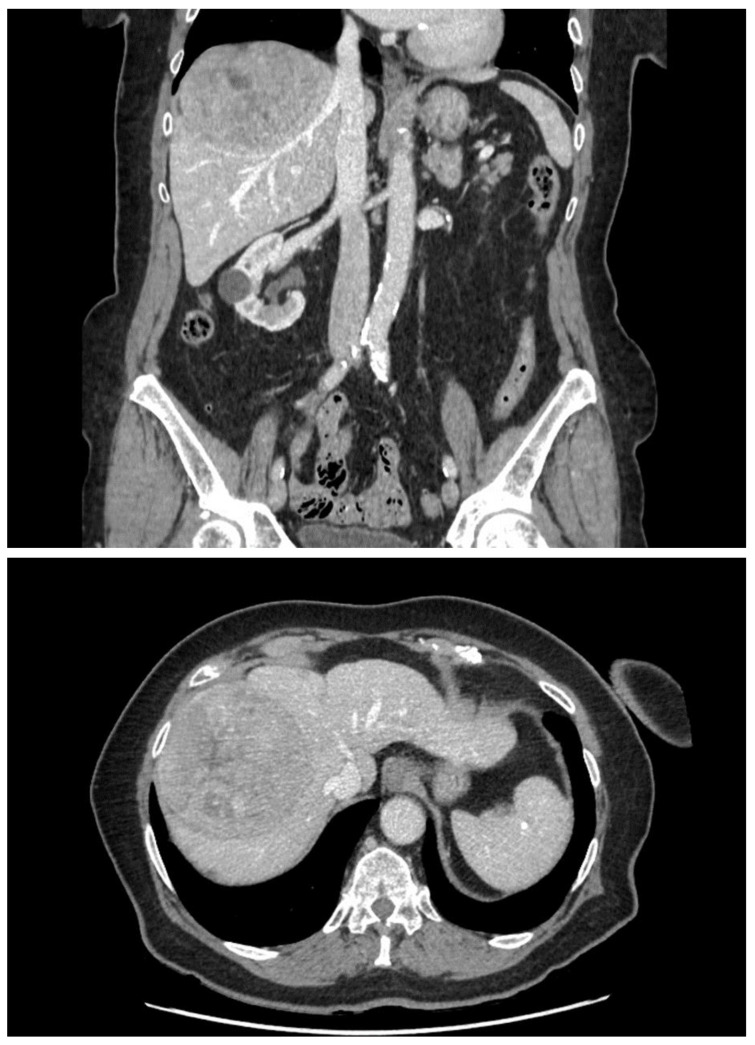
CT of the abdomen before TACE showing extensive tumor of liver segments 7 and 8.

**Figure 2:**
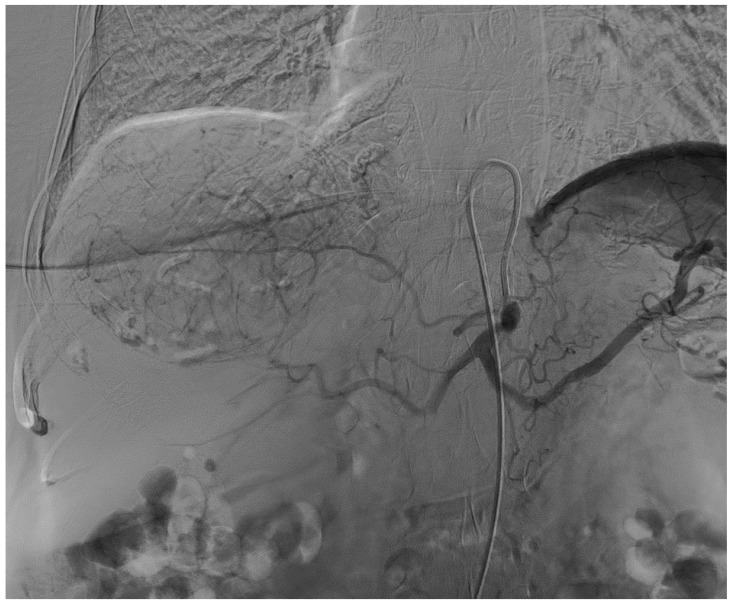
Pre-embolization digital subtraction celiac trunk angiogram showing a hypervascular tumor supplied by branches of the right hepatic artery.

**Figure 3:**
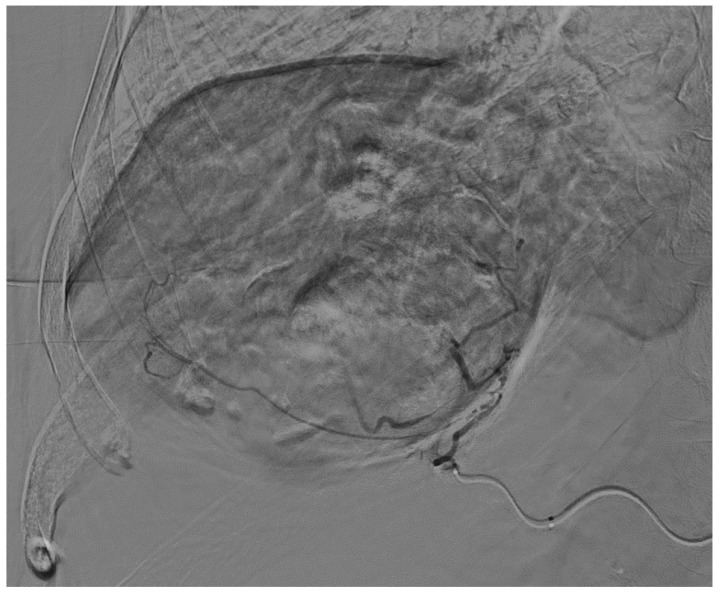
Post-embolization selective injection through the microcatheter showing significantly reduced tumor vascularity.

**Figure 4:**
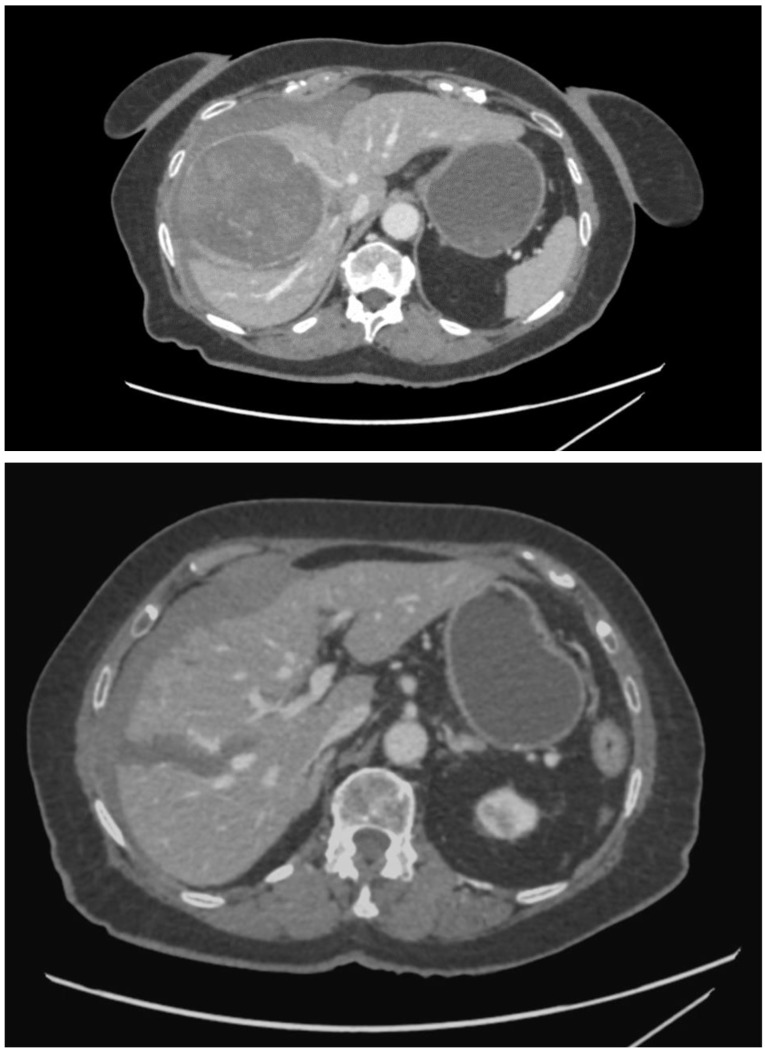

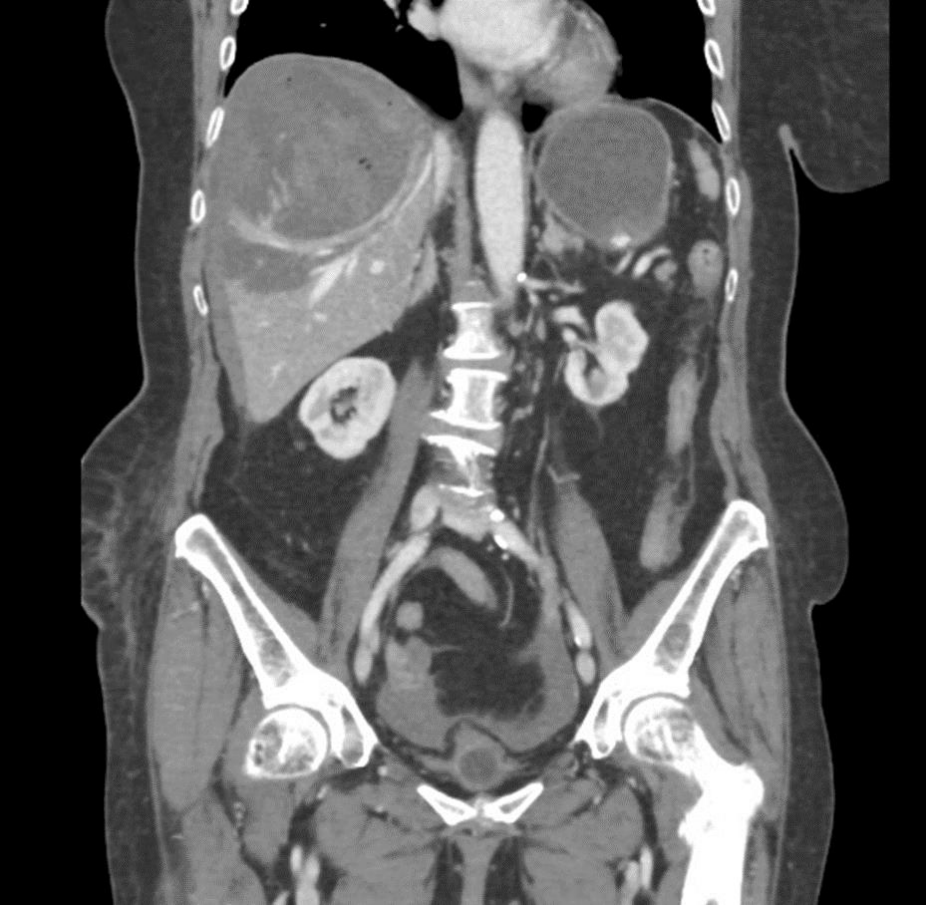
CT of the abdomen after TACE showing devascularization of the tumor, liver rupture, and hemoperitoneum.

An 82-year-old Caucasian woman with a past medical history of type 2 diabetes mellitus, arterial hypertension, chronic kidney disease, and peripheral arterial disease was admitted for an elective TACE of a sizeable histologically confirmed HCC (95 × 95 × 73 mm) of a normal liver with capsular location and numerous small satellite metastases (Figure 1). The tumor was determined as non-operable because of its size and the high periprocedural risk of complications. Liver transplantation was not considered because of the age of the patient. We offered the patient systemic chemotherapy, which she declined. TACE was established as a destination treatment through joint decision-making. During admission, the patient was well and had stabilized chronic diseases. An echocardiographic examination was carried out beforehand with normal findings. After administration of local anesthesia in the right groin, a 6 French sheath was introduced into the right common femoral artery. Selective catheterization of the celiac trunk was performed with the use of a 6 F guiding catheter followed by superselective catheterization of the right hepatic artery using a Direxion 2.4 F microcatheter (Boston Scientific, Natick, MA, USA) (Figure 2). The doxorubicin-eluting HepaSphere beads (Merit Medical, South Jordan, UT, USA) of sizes 120–240 µm (2 vials, 25 mg per vial) and 200–400 µm (2 vials, 25 mg per vial) were infused via superselective infusion of the feeding artery. Additional embolization of the target vessel with 2 mL of 300–500 mm unloaded Embosphere microspheres and 2 mL of 500–700 mm microspheres (BioSphere Medical, Rockland, MA, USA) was performed until partial stasis was achieved (Figure 3). The same position of the microcatheter in the common trunk of the feeding artery was used for embolization. During the procedure, preventive antibiotics and fluids were administered. After 20 h, the patient developed signs of shock with a drop in hemoglobin. Because hemorrhagic complication was anticipated, a CT scan was performed, which showed laceration of S8 and S5 liver segments up to the inferior vena cava and hemoperitoneum (Figure 4). Since the CT scan did not show signs of active extravasation of the contrast medium, TAE was not considered. The patient was indicated for acute open surgical revision of the abdomen. During the laparotomy, a large, perforated tumor of the right liver lobe with metastasis and hemoperitoneum was found. Local hemostatic treatment and tamponade to the perihepatic area were applied. Even though the bleeding was successfully stopped, the patient developed progressive multi-organ failure and passed away after a few days.

## 3. Discussion

Primary liver cancer is the sixth most common cancer worldwide, with HCC the most common type. Although cirrhosis is usually the scenario in which HCC develops, 20% of HCCs have been found to occur in non-cirrhotic livers [3]. The age distribution of non-cirrhotic HCC is bimodal, peaking in the second and seventh decades of life [4]. Significant information regarding HCC that develops in non-cirrhotic livers is generally lacking. Additional investigation is required to examine the epidemiology of non-cirrhotic HCC [5]. The risk factors for HCC in non-cirrhotic livers are diabetes mellitus, alcohol, chronic hepatitis, and smoking.

The incidence of spontaneous rupture of HCC varies from 3 to 26% of patients, with a reported mortality rate of 32%. The following findings have been reported as risk factors for spontaneous rupture of HCC: male sex, huge tumor size (reported diameter more than 5–7 cm), the tumor’s placement within the capsular tissue, its protrusion and exophytic tumoral growth (protruding more than 1 cm from the surface), tumor location (segments II, III, IV B, and VI), portal vein system thrombosis, cirrhosis, positive HBsAg as an independent risk factor, and the obstruction of the feeding artery [6,7].

Intra-peritoneal bleeding is a severe complication of any invasive procedure. The reviewed literature describes cases of gastric and duodenal ischemia [8] and gastric ulcer bleeding [9,10] associated with TACE. Two studies discussed the complications of TACE. Authors Xia et al. studied severe and rare complications of TACE. A total of 1348 cases of liver cancer were retrospectively evaluated, and 3 patients were found to have rupture of the tumor. Other complications were liver abscesses, hepatic artery spasms, hepatic artery occlusion, bilioma, and acute renal failure [11]. Another retrospective single-center study from authors Marcacuzco Quinto et al. documented sixteen complications during 322 procedures, from which only one was liver rupture [12]. As mentioned above, liver rupture after TACE is a very rare complication [13]. Table 1 reports all patients with the rupture of HCC after TACE, which were searched on the PubMed database. The mechanism behind rupture and bleeding after TACE is not understood. Suggested mechanisms are worsened phagocytic activity of macrophages, which results in vascular injury [14] and disturbances in the proliferation of elastin and collagen fibril degradation or inflammation secondary to chemotherapeutic agents [15]. HCC is usually a hypervascular tumor with many abnormal vessels. The risk of rupture following TACE may be increased by the interaction of variables such as reactive tissue edema linked to TACE and local vasculopathy linked to cancer. 

Predisposing risk factors for HCC rupture after TACE are comparable to those for spontaneously occurring HCC rupture. Risk factors specific to rupture after TACE are the first session of TACE and TACE performed without previous hepatic resection [16]. Authors Zhao et al. classified rupture of HCC following TACE into three categories based on their relationship with the liver capsule—type I (less than 30% of tumor cambered outward), type II (more than 30% and less than 50% of tumor cambered outward), and type III (more than 50% of tumor cambered outward) [17]. Based on this classification, they described in their retrospective analysis five patients with type III ruptured HCC compared to one patient with type I and two patients with type II ruptured HCC. All patients with type III ruptured HCC died. The authors suggested a new classification system for ruptured HCC and showed that patients with type III HCC are at increased risk of rupture. This information is necessary to establish periprocedural risk and personalize management.

The reported incidence of HCC rupture after TACE varies from 0.4 to 0.9% [18]. A study by authors Sun et al. described five cases of HCC rupture after TACE from a total of 1005 HCC patients [19]. Rupture of HCC after TACE was reported to have occurred 6 h after the procedure up to 7 months. The study by Jia et al. showed the interval between HCC rupture and the TACE procedure to be 2–17 days [15]. Another study of five cases showed an interval of 16 h to 7 months [19]. Based on Table 1, the time between TACE and ruptured HCC varies between 6 h and 7 months. The survival rate decreases with a shorter interval between TACE and rupture. As presented in Table 1, the literature describes 33 cases, 27 men with an average age of 59.8 years. In 24 patients, the tumor was located in the right lobe, 3 patients had tumors located in the left lobe, and 6 patients had tumors located in both lobes, with an average size of 9.4 cm. Ruptured HCC following TACE is a severe life-threatening complication; 18 patients mentioned in our search died.

It is difficult to determine correct management because of the significant difference in the time between HCC rupture and TACE. Further studies with larger numbers of patients are needed to determine proper management. It has not yet been determined how long the patient needs to be monitored. It is also necessary to approach the patient individually and evaluate the risk factors of HCC rupture.

Currently, transarterial embolization (TAE), followed by elective surgery, is preferred as the most effective treatment in patients with spontaneous HCC rupture [7]. On the other hand, a very recent analysis showed better prognosis in the group of patients with HCC and tumor rupture at the time of diagnosis who underwent surgical resection in comparison with non-surgery treatment modalities [20]. Another meta-analysis showed TACE/TAE outcomes comparable to emergency surgery regarding successful hemostasis and one-year survival [18].

The appropriate treatment of TACE-associated rupture of HCC is discussed and was found to be poorly described. As shown in Table 1, only three patients after TACE-associated ruptured HCC underwent a laparotomy, and every patient died. Conservative treatment was provided to 12 patients (8 died; 66.67%), and 18 underwent TAE (6 died; 30%). Some authors have suggested that emergency selective arterial embolization should be the standard if the patient cannot be managed conservatively—i.e., the patient is not hemodynamically stable [2,19]. More retrospective and prospective studies are crucial to determine the best evidence-based treatment choice for patients with ruptured HCC after TACE. We chose open laparotomy as the treatment option in our case report because of the severity of hemorrhagic shock and the difficulties in identifying the specific bleeding site, which made TAE unfeasible. 

A special category of patients undergoing TACE is patients over 80 years old. A recent study involved only patients with HCC over 80 years old who received TACE either as their first-line treatment or as salvage therapy for recurrence. A total of 86 patients were included and met the inclusion criteria. The study indicated the safety and efficacy of TACE for octagenarians with HCC—the response rate was 95.3% and there was no 30-day mortality, in-hospital mortality, and treatment-related deaths. One patient developed bleeding from the tumor after TACE (the tumor was located subcapsularly) [21]. 

**Table 1:** Previously reported cases of patients with ruptured HCC following TACE.

Author	Sex (Age)	History	Tumor (Location and Size)	Time between TACE and Rupture	Treatment following Rupture	Outcome
Yeh (2002) [22]	Male (45)	HCV and cirrhosis	Left and right lobe (segment IV–VIII), size NA	2 months	Laparotomy	Died
Battula (2007) [23]	Male (61)	Cirrhosis	Right lobe, 11 cm	2 days	Laparotomy	Died
	Male (69)	Cirrhosis	Right lobe, 13 cm	24 days	Conservative	Alive
Reichmann (2009) [24]	Male (53)	Cirrhosis and HBV	Right lobe (segment VII, VIII), 6 cm	6 h	Laparotomy	Died
Reso (2009) [13]	Male (90)	-	Right lobe, close to the liver capsule	4 h	Conservative	Died (respiratory failure)
Nawawi (2010) [25]	Male (66)	Cirrhosis	NA	NA	NA	Died
Sun (2010) [19]	Female (28)	-	Right lobe, 13 cm	1 month	TAE	Alive
	Female (42)	-	Right lobe, 11 cm	3 days	TAE	Alive
	Female (83)	-	Right lobe, 14 cm	5 months	TAE	Died
	Male (51)	-	Right lobe, 7 cm	16 h	TAE	Alive
	Male (47)	-	Right lobe, 10 cm	7 months	TAE	Alive
Ritter (2011) [26]	Male (74)	Cirrhosis	Left lobe (segment III, IVb), 16 cm	14 h	Conservative	Died
Bruls (2011) [27]	Male (78)	Cirrhosis	Left lobe (segment II, IV), 7 cm	3 weeks	Conservative	Died
Park (2011) [28]	Male (52)	Cirrhosis	Right lobe (segment VII), 12.3 cm	30 days	Conservative	Died
Jia (2013) [15]	Male (45)	Cirrhosis and HBV	Right lobe, 9 cm, near the liver capsule	10 days	Conservative	Died
	Male (61)	-	Right lobe, 13 cm, near the liver capsule	6 days	TAE/conservative *	Alive
	Male (53)	Cirrhosis and HBV	Right and left lobe, 11 cm, near the liver capsule	7 days	Conservative	Died
	Male (57)	HBV	Right and left lobe, 14 cm, near the liver capsule	9 days	TAE/conservative *	Alive
	Male (64)	-	Right and left lobe, 16 cm, near the liver capsule	17 days	TAE/conservative *	Alive
	Male (67)	-	Right and left lobe, 16 cm, near the liver capsule	13 days	TAE/conservative *	Alive
Singh Bhinder (2015) [2]	Male (67)	HCV and cirrhosis	Right lobe (segment VII), 4 cm	1 day	TAE	Alive
Tu (2016) [29]	Male (45)	HBV and cirrhosis	Right lobe, 9 cm	10 days	Conservative	Died
Nishida (2018) [10]	Male (81)	Cirrhosis	Left and right lobe (segment II, VI, VIII), 6.5 cm and three lesions less than 2 cm, superficial	5 weeks	Conservative	Alive
Gala (2020) [16]	Female (76)	AIH cirrhosis	Right lobe, 6 cm	12 h	Conservative	Died (she developed HAP)
Zhao (2021) [17]	Male (63)	-	Right lobe (segment VIII), 3.2 cm	16 h	TAE	Alive
	Male (70)	-	Right lobe (segment V), 4.2 cm	40 h	TAE	Died
	Female (44)	-	Right lobe (segment VIII), 8.8 cm	7 days	TAE	Died
	Male (48)	-	Right lobe (segment VI), 9.1 cm	4 days	TAE	Died
	Male (57)	-	Right lobe (segment V), 5.2 cm	13 days	TAE	Died
	Male (60)	-	Right lobe (segment VI), 7 cm	10 days	TAE	Died
	Female (58)	-	Right lobe (segment VIII), 7.3 cm	3 days	TAE	Alive
	Male (51)	-	Right lobe (segment VII), 8.6 cm	5 days	TAE	Alive
Parthasarathy and Khan (2024) [30]	Male (66)	Alcohol-relater liver cirrhosis	Right lobe (segment VIII), 3.3 cm	30 h	TAE	Alive

Note—* This is in reference to the fact that from the 4 patients in the study of Jia et al., 2 patients received TAE and 2 patients had conservative treatment (medication for pain relief, hemostasis, and energy input and rest) [15]. AIH—autoimmune hepatitis, HAP—hospital-acquired pneumonia, HCV—hepatitis virus C, HBV—hepatitis virus B, and NA—not applicable.

## 4. Conclusions

This case report aims to draw attention to a rare but severe complication of HCC rupture after TACE. Despite TACE’s recognized efficacy as a minimally invasive treatment option for liver cancer, particularly in non-operable cases, the occurrence of post-procedural complications such as liver rupture necessitates careful consideration. The best therapeutical option for the rupture of HCC after TACE has not yet been established. It is important to recognize patients with a high risk of rupture of HCC following TACE based on the risk factors and consider potential treatment options and post-procedure monitoring beforehand. More data and studies are vital to determine the best evidence-based approach for patients with this rare complication. Future studies should aim to establish a clearer consensus on the most effective treatment modalities, taking into account the unique risk profiles of individual patients. As the population ages, the safety and efficacy of TACE in older patients, particularly those over 80, also warrant additional exploration to ensure optimal outcomes. This manuscript not only contributes to the existing literature on the complications associated with TACE but also emphasizes the critical need for ongoing research in this area of research.

## Data Availability

The data presented in this study are available on request from the corresponding author.
